# Comparison of growth performance and rumen metabolic pathways in sheep and goats under the same feeding pattern

**DOI:** 10.3389/fvets.2023.1013252

**Published:** 2023-02-09

**Authors:** Xueyan Lin, Lin Ju, Qianjin Cheng, Yue Jiang, Qiuling Hou, Zhiyong Hu, Yun Wang, Zhonghua Wang

**Affiliations:** College of Animal Science and Veterinary Medicine, Shandong Agricultural University, Taian, Shandong, China

**Keywords:** sheep, goats, rumen metabolism, ruminal microorganisms, different strains

## Abstract

Diet and species are important factors affecting the rumen microbiota, with roughage stimulating rumen development and concentrate feeds being broken down by the decomposition of Ruminal flora to provide the organism with a large amount of energy. This study aimed to explore the effects of host and dietary factors on rumen flora composition and diversity, as well as on host metabolism. The study reports the research conducted on 5-month-old male Small-tail Han sheep and 5-month-old male Boer goat, each with an average weight of 33.87 ± 1.70 kg. Five animals of each species were divided into two groups, namely, the S group (Small-tail Han sheep) and the B group (Boer goat). The experiment was carried out in two various periods, namely, X and Y for groups S and B, respectively. The rations were fed with concentrate-to-roughage ratios of 3:7 and 5:5, respectively. Growth performance was measured by the weight increase index. The results showed that, under the same raising conditions, the ratio between body weight increases and the amount of feed was lower in the S group than in the B group, but the differences were not significant. According to the analysis of the apparent digestibility ratio of nutrition ingredients, the XS group had a significantly higher apparent digestibility ratio for acid detergent fiber than the XB group (*p* < 0.05). Even though the analysis of rumen fermentation parameters showed that the rumen pH has no significant differences between the XS and XB groups, it was significantly lower in the YS group than in the YB group. The XS group contained a significantly lower content of total volatile fatty acids than the XB group (*p* < 0.05). Analysis of the 16S rDNA sequencing results revealed that, compared to the B group, the S group was highly enriched with the following bacteria: Proteobacteria, γ-proteobacteria, Aeromonadales, and Succinivibrionaceae. Thus, the host species affected the abundance and diversity of rumen bacteria. Feed utilization efficiency of Small-tail Han sheep was higher than Boer goats, which might be specifically associated with Succinivibrionaceae. The results from this study show that animals belonging to the same family but different genera and species can differ in metabolic pathways even when they are provided with the same animal feed.

## 1. Introduction

The complexity of the rumen microbial community network and its relevance to host fermentation metabolism determine that the diet is an important factor influencing the composition and structure of the rumen microbial community. A study was conducted to classify Nubian goats into high phosphorus (HP) digestibility phenotypes and low phosphorus (LP) digestibility phenotypes, and it was found that the structure of the gastrointestinal microbiota of goats with different true digestibility of phosphorus in the ration (TDP) was significantly different. Ruminal and wrinkled stomach microorganisms had the greatest effect on host TDP, followed by other segments of the gastrointestinal tract (1). The addition of different concentrations of branches and leaves trimmed from tea tree (BLTT) to the diets of 32 Nanjiang Yellow goats revealed that the addition of BLTT improved the antioxidant capacity, rumen fermentation characteristics, gastrointestinal development, and overall growth performance of the goats (2). On studying the relationship between the true digestibility of calcium (TDC) in goat rations and gastrointestinal microorganisms, it was found that the structure of goat gastrointestinal flora was related to TDC. In addition, some gastrointestinal bacteria, such as Prevotella rumen, are favored to improve the true digestibility of dietary calcium in the host (3). Feeding soy protein concentrate diets increase the number of short-chain fatty acid-producing microorganisms in the gut of pullets (4).

The host genome is also one of the predominant factors influencing the intestinal microbiota. Under the same raising environments and the same feeding regiments, the digestive system of different species normally contains different microbial communities; moreover, within the same species of different genetic lines, the microbial community composition in the digestive tracks also varies significantly. This suggests that the microbial system of the host's digestive tract is influenced by its genome (5).

The host species is an important factor affecting rumen microbiota. In ruminant animals, 50–70% of protein supplies and up to 70% of energy needs are provided by the rumen microbial activities. A study was performed on inter-transplant intestine microbes in pre-disinfected mice and zebrafish. The results showed that the receiver intends to develop an intestine microbiota, in terms of both the species composition and the relative abundance, more similar to the donor (6). Raising lizard species requires a very strict environment as they have unique intestine microbial communities. Farm-raised lizards, which was separated from the mother, contained 34.3% of the same intestine bacteria as the parent, indicating that the intestine bacterial species was influenced by genetics (7). Under the same growth environment, different species of leech were found to develop very different intestine microbial populations (8). An increasing number of studies support the idea that host species and their genetic background are an important factor, in addition to feed, influencing microbial communities through microbial–host interactions.

For Ruminant animals, the rumen contains a very large number of microbes playing a major role in the breakdown of feed components that mammalian animals can utilize them. During the degradation process of animal feed, the microbial proteins, vitamins, and volatile lipids can all be converted into nutrients for the host animals. Products from microbial physiological activities provide nutrients and various regulatory molecules that are essential for the host animals. Through participation in many physiological and metabolic processes of the host, the microbiota influences the health and growth of the host animals. The addition of dimethylglycine to broiler diets can alleviate the intestinal barrier damage produced by heat stress by affecting the microbial metabolism of the intestinal-brain axis (9). The microbiota in animal digestive tracks affect metabolites of short-chain lipids, the integrity of the intestinal barrier, the neurosystem (neurotransmitter), neuroendocrine pathways (hormones), and others, and they are involved in the immunity, metabolism, infection, neurosystem, behavior, and psychopathology of the host (10). The intestinal bacteria and the host have a balanced mutual-benefit relationship (11). Understanding microbiota–host interactions will help improve the productivity of the animals. This study was conducted to determine the response of two host species to two diets providing different ratios of concentrate feed/crude feed. The 16S rDNA sequencing and metabolism analysis were used to investigate the effects of host species on rumen microbiota and the endogenous metabolites accumulated in the host animals.

## 2. Materials and methods

### 2.1. Experimental instruments

Muffle furnace (SGM·M16/10, SIGMA), crusher (DFT1000, China Shenzhen Leitong), Kjeldahlnitrogen analyzer (ATN-300, Shanghai HongJi Instrument), UV spectrophotometer (Shimadzu,UV-1780), centrifuge (TD5A, Changzhou Runhua Electric Co.), ruminal fluid collector (MDW15, Shanghai Model Organisms Center, Inc.), pen-type pH meter (BPHPOCKET-C, BELL Analytical Instruments), automatic biochemical analyzer (type 7020, HITACHI), gas chromatography–mass spectrometer (ISQ7610-AEI, Thermo Fisher).

### 2.2. Experimental animals and experimental design

This experiment was conducted at the Experimental Station of Animal Husbandry Science and Technology, Shandong Agricultural University, Taian, China. The experimental animals were 5-month-old male with an average body weight of 33.87 ± 1.70 kg. The Small-tail Han sheep (S group) and Boer goat (B group) were tested each with five animals; they were fed with two different diet formulas of concentrate-to-crude feed ratios of 3:7 and 5:5, respectively. The experimental pre-feeding period was 7 days, and the formal period was divided into periods X (28 days) and Y (28 days) for a total of 56 days, with a concentrate-to-coarse ratio diet ratio of 3:7 in period X (further divided into XS and XB groups) and a concentrate-to-coarse diet ratio of 5:5 in period Y (further divided into YS and YB groups). The amount of daily feed was estimated at 3.5% of body weight. Animals were raised in the same environments during the experimental period.

### 2.3. The experimental diet

The formula of experimental diets was designed based on the National Research Council (NRC) animal feed standards (2001). The animal feed was in the form of total mixed ration pellet feed. At the beginning of each experimental period, the number of grams of food given in time was weighed again, feeding was done in two equal portions of ratios 8:00 and 17:00 daily, and the drinking water used was fresh and sufficient. Each animal was given a licking salt block to meet mineral nutrient requirements. The diet ingredients and nutrient composition are summarized in Table 1.

**Table 1:** Ingredients and nutrient composition of diets fed to the animals (dry matter basis).

**Item**	**Diet**	
	**Concentrate ratio, 3:7**	**Concentrate ratio, 5:5**
**Ingredients (% DM)**		
Corn	18.5	20.5
Soybean meal	6	10.45
Bran	4.5	19
Peanut vine	36	20.5
Alfalfa Hay	33	27
CaHPO_4_	0.5	0.25
Limestone	0	0.8
NaCl	0.5	0.5
Premix1	1	1
Total Nutritional ingredient Dry matter (DM), % of Diet	100 92.00	100 90.44
Crude protein (CP), % of DM	13.08	15.15
Neutral detergent fiber (NDF), % of DM	39.30	37.96
Acid detergent fiber (ADF), % of DM	24.76	23.42
Ether extract (EE), % of DM	1.23	1.29
Non-fiber carbohydrates (NFC)^1^	35.47	37.16
Ca, % of DM	1.18	0.99
P, % of DM	0.35	0.44

1, NFC = 100 - (% NDF + % CP + % EE + % Ash.).

Premixed diet ingredients: VA 1,000 KIU/kg; VD 3,250 KIU/kg; VE 2,400 mg/kg; nicotinic acid 2,000 mg/kg; Fe 2,000 mg/kg; Mn 3,000 mg/kg; Cu 3,000 mg/kg; Zn 14,000 mg/kg; Se 100 mg/kg; I 180 mg/kg; Co 40 mg/kg.

### 2.4. Sample collection and analysis

A total of 200 g of samples were taken from the diet using the point-centered quarter method. Samples were dried at 65°C until constant weight. After air-drying, the samples were weighed and then ground into the 1-mm-sized pellet before being used for routine animal feed analysis.

Samples were also collected using the point sampling method where samples were collected every 8 h using acid insoluble ash (AIA) as the endogenous indicator. Two days before the end of each experimental phase, feces were collected and oven-dried at 65°C until constant weight is achieved. After the removal of surface debris, the samples were ground into fine powder. The total amount of feces was calculated after calibration against the AIA values.


$$\begin{array}{l} Totalfeces=\left(A1\times B1\right)/C, \end{array}$$

where A1 is the amount of feeding (kg); B1 is the percentage of AIA in the feed (%); and C is the amount of AIA in feces (%). There was a minimal, negligible amount of leftover feed; therefore, it was not taken into account in the analysis.

Ruminal fluid was collected through a gastric tube rumen sampler 3 h after morning feeding every 7 days in each period and filtered through 2 layers of gauze. Ruminal fluid samples were obtained by filtering through two layers of gauze, and the pH value was measured with a pen pH meter immediately after ruminal fluid collection. The rumen fluid samples were then divided into one 5-ml lyophilizer tube and four 2-ml lyophilizer tubes and stored at −80°C. The last lyophilized 5-ml portion of the last sample was used for volatile fatty acid determination and a 2-ml portion was used for volatile fatty acid determination. The remaining samples were used for freezing backup.

At the end of the preparatory period as well as after each feeding phase, the body weight of the animals was measured using an electronic bench scale. The total RNA was extracted from the rumen fluid; primers were designed against conserved regions of the 16S dRNAs; and a sequencing index was added to the primers. After polymerase chain reaction (PCR) amplification, the PCR products were purified and quantified before the preparation of sequencing libraries. The dRNAs were sequenced on an Illumina HiSeq 2500. Sequence-based clustering was conducted using the 97% similarity threshold.

## 3. Results

### 3.1. Increases in body weight of experimental animals

As shown in Table 2, raised under the same conditions, it was found that both groups of animals had no significant differences (*p* > 0.05) in the initial body weight, average daily increase in body weight, daily food consumption, and food/body weight ratio. The average daily weight gain of the whole experiment (1–56 days) showed a trend of higher difference between groups S and B (0.05 < *p* < 0.1). The food/body weight ratio was the amount of animal feed required per 1,000-g increase in body weight; the smaller the number, the higher efficiency of animal productivity. The food/body weight ratio in the S group was lower than that in the B group.

**Table 2:** Growth performance of sheep and goats fed on the same diet.

**Item**	**Total number of days of experiment**	**Group**		***p***
		***S***	***B***	
**Body weight, kg**				
	0	34.44 ± 1.00	33.29 ± 1.09	0.46
	27	38.44 ± 1.16	36.61 ± 1.29	0.32
	54	44.28 ± 1.53	41.34 ± 1.61	0.22
**Average daily growth ADG, kg/day**				
	X phase (1–27)	0.15 ± 0.01	0.12 ± 0.01	0.23
	Y phase (28–54)	0.22 ± 0.02	0.18 ± 0.01	0.11
**Daily dry matter intake**, **DMI, kg**				
	X phase (1–27)	1.21 ± 0.04	1.17 ± 0.04	0.46
	Y Phase (28–54)	1.35 ± 0.04	1.28 ± 0.05	0.32
**Feed-to-gain ratio** **F/G**				
	X Phase (1–27)	8.33 ± 0.61	10.05 ± 1.32	0.27
	Y Phase (28–54)	6.35 ± 0.42	7.45 ± 0.40	0.09

### 3.2. Apparent digestibility of nutrient ingredients

As shown in Table 3, the apparent digestibility of crude protein, neutral detergent fiber, and acid detergent fiber from the two groups of animals was presented. The apparent digestibility of acid detergent fiber from the XS group is significantly higher than that from the XB group (*p* < 0.05).

**Table 3:** Apparent digestibility of nutrients by sheep and goats fed on the same diet.

**Item**	**Group**				**Group**			
	**XS**	**XB**	**SEM**	***P***	**YS**	**YB**	**SEM**	***p***
**Crude protein (CP)**								
Feed Intake, kg/day	0.16	0.15	0.01	0.46	0.20	0.19	0.01	0.32
CP of feces, kg/day	0.05	0.05	0.01	0.79	0.05	0.05	0.00	0.85
Apparent digestibility, %	70.20	69.97	2.78	0.94	74.84	73.14	1.33	0.23
**NDF**								
Intake, kg/day	0.47	0.46	0.02	0.46	0.51	0.49	0.02	0.32
NDF of feces, kg/day	0.20	0.20	0.02	0.90	0.24	0.26	0.02	0.40
Apparent digestibility, %	58.68	56.49	3.45	0.54	52.94	46.88	3.48	0.12
**ADF**								
Intake, kg/day	0.30	0.29	0.01	0.46	0.33	0.32	0.01	0.32
ADF of feces, kg/day	0.15	0.17	0.01	0.18	0.18	0.19	0.02	0.43
Apparent digestibility, %	50.13	41.90	3.41	0.04	45.84	38.39	4.73	0.15

### 3.3. Analysis of rumen fermentation parameters

As shown in Table 4, the pH of rumen fluid in the YB group is significantly higher than the YS group; XB also has higher contents of total volatile fatty acids and acetic acids than the XS group (*p* < 0.05). There were no significant differences in the contents of acetate and propionate between the two groups of animals throughout the treatment periods (*p* > 0.05).

**Table 4:** Rumen fermentation parameters by sheep and goats fed the same diet.

**Item**	**Group**		***p***	**Group**		***p***
	**XS**	**XB**		**YS**	**YB**	
Mean pH	6.55 ± 0.10	6.58 ± 0.10	0.83	6.32 ± 0.05	6.51 ± 0.03	0.01
TVFA, mmol/L	102.36 ± 0.92	105.91 ± 0.76	0.02	95.15 ± 0.98	96.30 ± 1.06	0.45
Acetate	63.46 ± 0.98	67.12 ± 0.45	0.01	59.96 ± 1.07	60.45 ± 0.96	0.74
Propionate	19.42 ± 0.59	19.42 ± 0.36	0.10	17.00 ± 0.29	17.41 ± 0.32	0.37
Butyrate	13.04 ± 0.29	13.18 ± 0.35	0.77	12.35 ± 0.22	12.14 ± 0.10	0.41
Isobutyric acid	1.29 ± 0.05	1.28 ± 0.05	0.90	1.21 ± 0.04	1.28 ± 0.02	0.16
Valerate	2.57 ± 0.04	2.48 ± 0.17	0.63	2.31 ± 0.07	2.49 ± 0.04	0.06
Isovaleric acid	1.40 ± 0.07	1.25 ± 0.04	0.11	1.21 ± 0.09	1.42 ± 0.16	0.27
Caproic acid	1.18 ± 0.04	1.17 ± 0.04	0.85	1.11 ± 0.03	1.11 ± 0.04	0.89
A:P ratio	3.28 ± 0.11	3.46 ± 0.08	0.23	3.53 ± 0.11	3.48 ± 0.08	0.69

### 3.4. 16S rDNA sequencing

#### 3.4.1. Polymorphism of 16S rDNA

**Figure 1:**
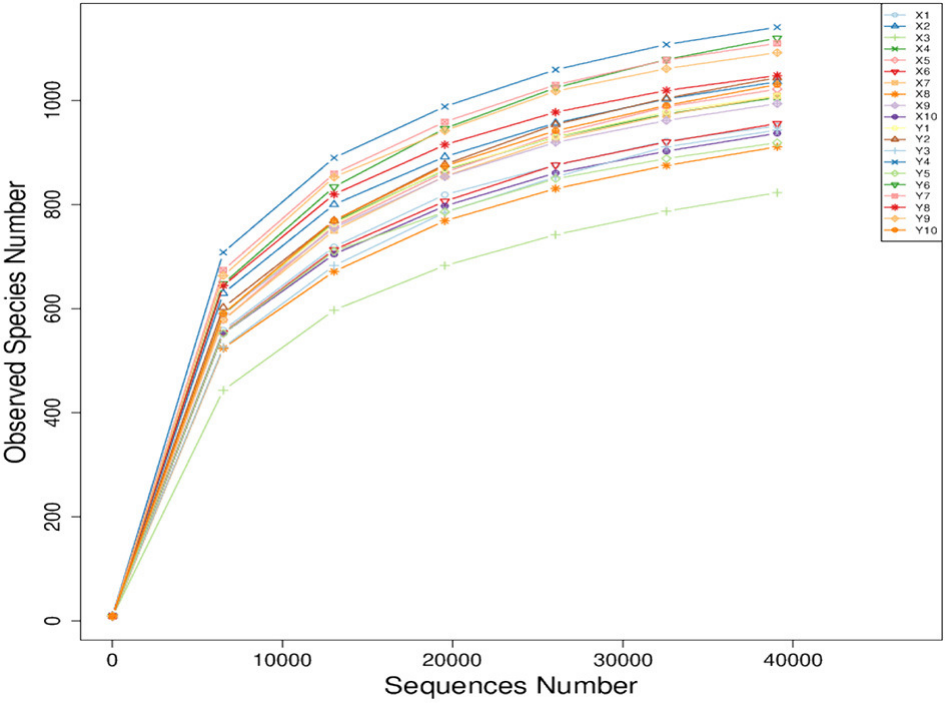
Sample-based rarefaction curve of observed species.

As shown in Figure 1, all the curves became smooth as the number of sequence reads increased, and the number of operational taxonomic units (OTU) constructed using the sequencing reads have reached a plateau. These results indicate that there is enough sequencing depth to represent all the genomes from all the species in the samples.

#### 3.4.2. Annotation and classification of the species

**Figure 2:**
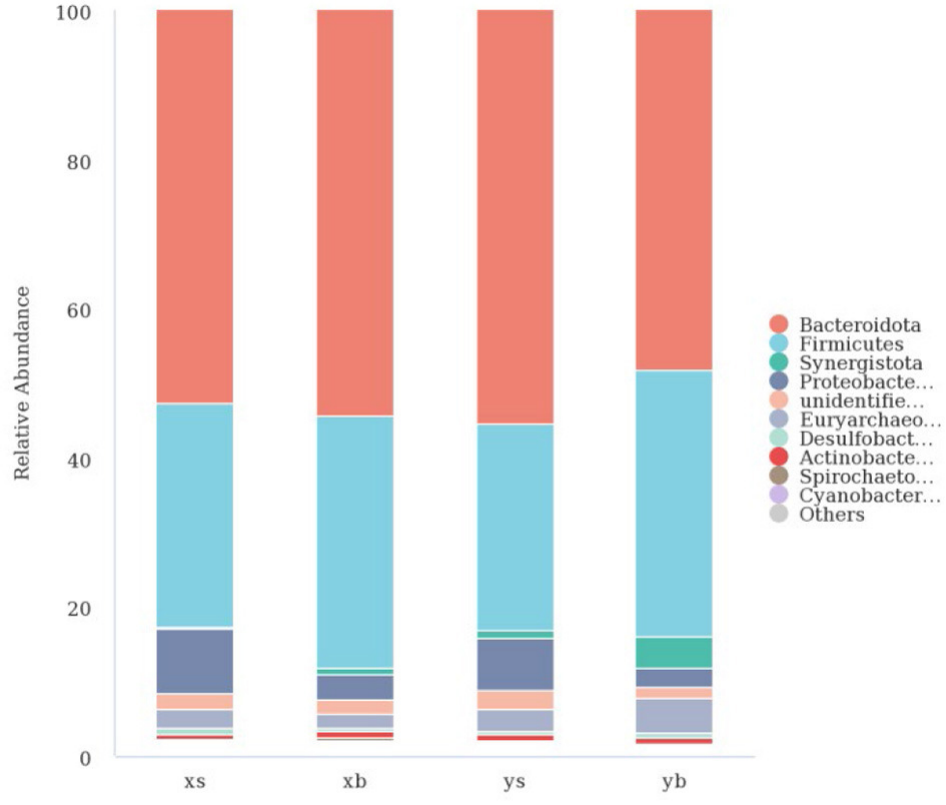
The taxonomic composition distribution in samples of the phylum level.

Species annotation was using the OUT sequences. The top 10 species with the highest abundance in each genus and family were selected from each treatment group; then, a column cumulative chart was generated to present the relative abundance of the bacterial communities. As shown in Figure 2, at the family level, the XS group is mainly composed of Bacteroidota (52.62%), Firmicutes (30.02%), Proteobacteria (8.68%), and Euryarchaeota (2.57%). The XB group is mainly composed of Bacteroidetes (54.43%), Firmicutes (33.73%), Proteobacteria (3.30%), and Euryarchaeota (1.81%). The YS group is mainly composed of Bacteroidetes (55.37%), Firmicutes (27.81%), Synergistota (1.02%), Proteobacteria (6.85%), Euryarchaeota (3.06%). The YB group is mainly composed of Bacteroidetes (48.30%), Firmicutes (35.67%), Synergistetes (4.25%), Proteobacteria (2.42%), and Euryarchaeota (4.79%).

**Figure 3:**
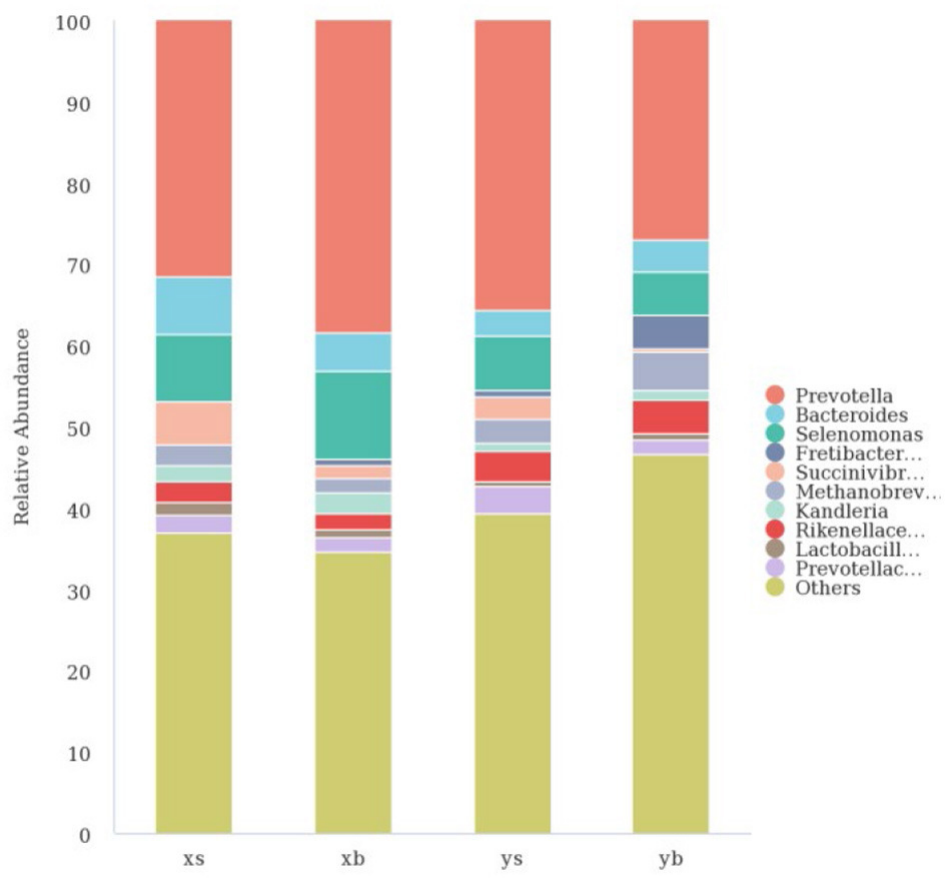
The taxonomic composition distribution in samples in the genus level.

As shown in Figure 3, at the genus level, the XS group contains mainly the following: Prevotella (31.48%), Bacteroides (7.09%), Selenomonas (8.17%), Succinivibrionaceae_UCG-001 (5.37%), Rikenellaceae_RC9 (2.53%), Methanobrevibacter (2.52%), Kandleria (1.98%), Lactobacillus (1.59%), and Prevotellaceae_UCG-001 (2.17%). The XB group contains the following major genera: Prevotella (38.36%), Bacteroides (4.76%), Selenomonas (10.80%), Succinivibrionaceae _UCG-001 (1.47%), Rikenella _RC9) (1.93%), Methanobrevibacter (1.77%), Kandleria (2.63%), Lactobacillus (1.07%), and Prevotellaceae_UCG-001 (1.74%). The YS group contains the following major genera: Prevotella (35.61%), Bacteroides (3.22%), Selenomonas (6.55%), Succinivibrionaceae_UCG-001 (2.71%), Rikenella _RC9 (3.88%), Methanobrevibacter (3.01%), and Prevotellaceae_UCG-001 (3.29%). The YB group contains the following major genera: Prevotella (26.94%), Bacteroides (3.99%), Selenomonas (5.37%), Fretibacterium (4.16%), Rikenella _RC9 (4.22%), Methanobrevibacter (4.74%), Kandleria (1.09%), and Prevotellaceae_UCG-001 (1.91%).

#### 3.4.3. Microbial species differ between the goat and sheep species

All identifiable bacteria were first classified in the order of phylum, class, order, family, and genus. Then, bacterial strains with counts >0.1% of the total rumen bacterial populations and showing significant differences between the S and B groups were selected. As described in Table 5, when comparing the XS and XB groups, the bacterial strains showing a significantly higher abundance in the S-treated (XS) group include Proteobacteria, γ-proteobacteria, Aeromonadales, Succinivibrionaceae, Succinivibrionaceae_UCG-001, and Succinivibrio.

**Table 5:** The difference of rumen fluid at the bacterium (XS-XB).

**Bacterium**	**Abundance% (XS)**	**SE (XS)**	**Abundance% (XB)**	**SE (XB)**	***P***
**Phylum**					
Proteobacteria	8.68	1.14	3.30	0.26	0.01
**Class**					
γ-Proteobacteria	8.64	1.14	3.25	0.27	0.01
**Order**					
Aeromonadales	8.41	1.12	2.98	0.32	0.01
**Family**					
Succinivibrionaceae	8.41	1.12	2.98	0.32	0.01
**Genus**					
Succinivibrionaceae_UCG-001	5.37	1.07	1.47	0.30	0.02
Succinivibrio	2.09	0.52	0.62	0.15	0.04

SE, standard error.

As shown in Table 6, when comparing the YS and YB groups, the S-treated rumen contained a higher abundance of bacteria including Proteobacteria, γ-proteobacteri, Aeromonadales, Succinivibrionaceae, Prevotellaceae_UCG-001, Shuttleworthia, Syntrophococcus, and Schwartzia.

**Table 6:** The difference of rumen fluid at the bacterium (YS-YB).

**Bacterium**	**Abundance% (YS)**	**SE (YS)**	**Abundance% (YB)**	**SE(YB)**	***P***
**Phylum**					
Proteobacteria	6.85	1.09	2.42	0.40	0.01
**Class**					
γ-Proteobacteria	6.79	1.10	2.35	0.40	0.01
**Order**					
Aeromonadales	6.56	1.10	2.03	0.46	0.01
Oscillospirales	3.46	0.75	5.52	0.33	0.049
RF39	0.42	0.10	1.27	0.23	0.02
**Family**					
Succinivibrionaceae	6.56	1.10	2.03	0.46	0.01
Ruminococcaceae	1.96	0.48	3.56	0.22	0.02
**Genus**					
Prevotellaceae_UCG-001	3.29	0.29	1.91	0.20	0.01
Quinella	0.52	0.12	1.61	0.38	0.04
Shuttleworthia	0.73	0.15	0.30	0.02	0.049
Syntrophococcus	0.18	0.04	0.04	0.01	0.03
Schwartzia	0.15	0.01	0.04	0.01	< 0.01

SE, standard error.

### 3.5. Metabolome analysis

#### 3.5.1. Metabolome of rumen fluids

**Figure 4:**
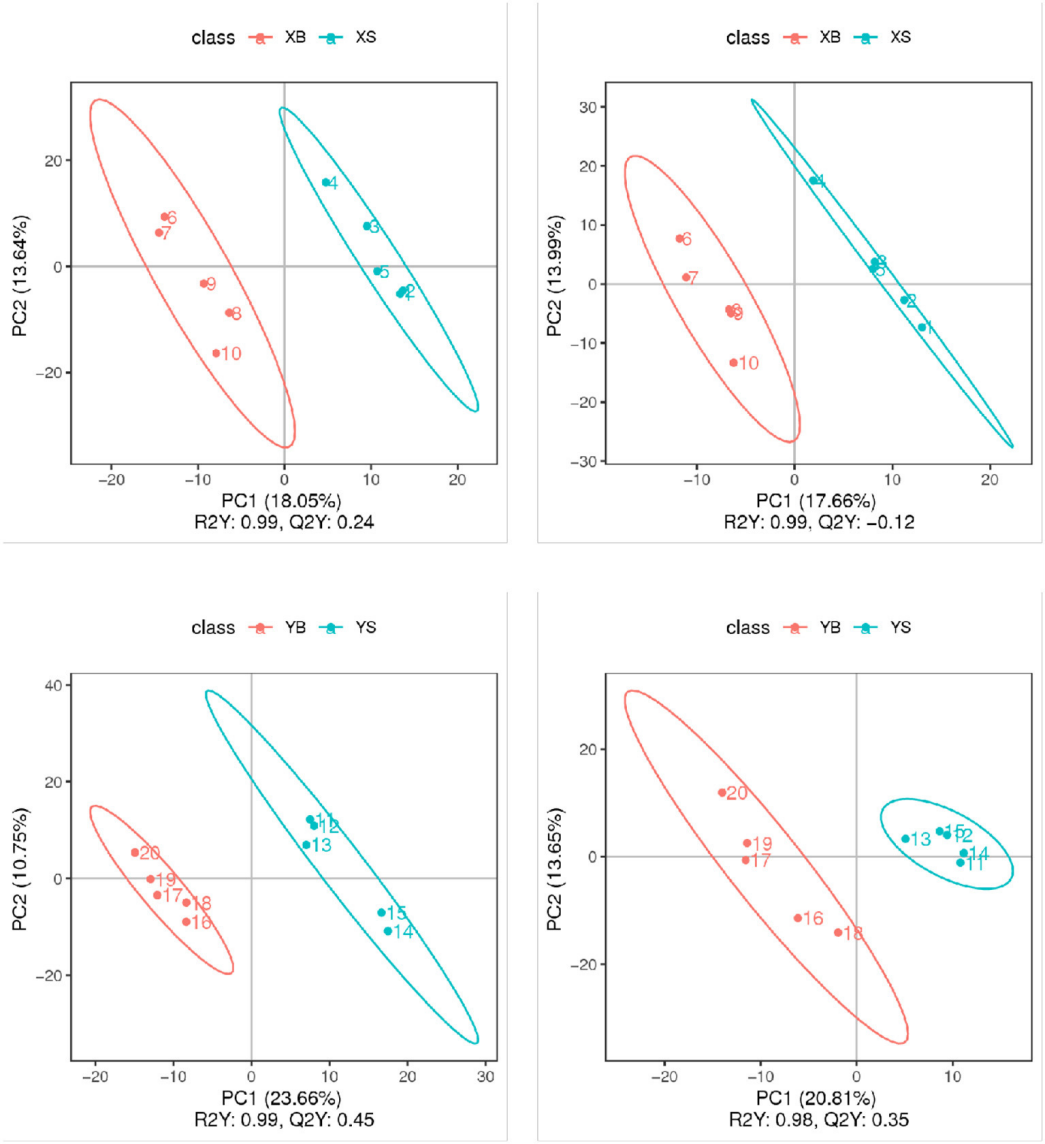
PLS-DA score plot (positive and negative ion mode). The *x*-axis indicates the scores of samples in the first components; the *y*-axis is the scores of samples in the second major component [R2Y indicates the explained variation in the model; Q2Y indicates the predictability of the PLS-DA model. A good model requires R2Y > Q2Y.

The partial least squares discrimination analysis (PLS-DA) models for each pair of treatment groups are shown in Figure 4. The replicate samples from the same treatment group were clustered in the same component; samples from different groups were clustered into different components. These results confirmed that the experimental data have very good replicability within groups, and there are big differences between groups.

#### 3.5.2. Differential metabolites in rumen fluid

The differential metabolites were selected based on the PLS-DA model (*p* < 0.05) using one-way ANOVA, and the fold change threshold of variable importance in projection (VIP) is >1. There were 94 significantly different metabolites in the XB group compared to the XS group, with 50 significantly upregulated and 44 significantly downregulated in the XS group. There were 131 significantly different metabolites in the YB group compared to the YS group, with 63 significantly upregulated and 68 significantly downregulated in the YS group. The differential metabolites were then enriched into the Kyoto Encyclopedia of Genes and Genomes (KEGG) metabolic pathways.

As shown in Table 7, when comparing the XS and XB groups, the S-treated rumen fluids contained significantly higher contents of the following metabolites: corticosterone and 4-methylcatechol; metabolites showing significant decreases include phenethylamine, guanine nucleotide, N2-(carboxyethyl)-L-arginine, 9S-hydroxy-10E,12Z,15Z-octadecatrienoic acid, indole, testosterone, malonic acid, and D-glucosamine-6-phosphate.

**Table 7:** Metabolites and their corresponding metabolic pathways (XS-XB).

**Metabolite**	**Metabolic pathways**	**Fold change**	***p***	**VIP**
Phenethylamine	Phenylalanine metabolism (map00360)	0.502	0.010	1.340
Guanosine monophosphate	Purine metabolism (map00230); cGMP-PKG signaling pathway (map04022)	0.416	0.011	2.567
Octopine	Arginine and proline metabolism (map00330); ABC transporters (map02010)	0.506	0.014	1.979
Corticosterone	Steroid hormone biosynthesis (map00140); Regulation of lipolysis in adipocytes (map04923); Aldosterone synthesis and secretion (map04925)	2.957	0.014	2.192
(9S)-(10E,12Z,15Z)-9-Hydroxy-10,12,15-octadecatrienoic acid	Alpha-Linolenic acid metabolism (map00592)	0.645	0.019	2.168
Indole	Tryptophan metabolism (map00380); Phenylalanine, tyrosine and tryptophan biosynthesis (map00400); Protein digestion and absorption (map04974)	0.086	0.029	1.978
Testosterone	Steroid hormone biosynthesis (map00140)	0.542	0.040	1.298
Malonic acid	Pyrimidine metabolism (map00240); Beta-Alanine metabolism (map00410)	0.412	0.003	1.781
4-Methylcatechol	Microbial metabolism in diverse environments (map01120)	3.056	0.036	1.958
D-Glucosamine 6-phosphate	Alanine, aspartate and glutamate metabolism (map00250); Amino sugar and nucleotide sugar metabolism (map00520); Insulin resistance (map04931)	0.454	0.044	1.728

Map is the number of reference path diagrams in KGGE.

As shown in Table 8, when rumen metabolites were compared between the YB and YS groups, the S-treated rumen (the YS group) led to significantly higher content of palmitoleic acid, cortisol, prostaglandin, J2, (9Z,11E,13S,15Z)-13-hydroperoxy-9,11,15-octadecatrienoic acid, 11,15-octadecadienoic acid, and ascorbic acid; the B treated rumen (YB group) contained significantly higher content of trimethyl lysine, taxifolin, adenylic acid, disodium inosinate, trigonelline, N6-acetyl-L-lysine, guanine nucleotide, spermidine, traumatic acid, xanthosine, spermine, 7α-hydroxycholesterol, D-ribofuranose,5-(dihydrogen phosphate, L-histidine, D-sedoheptulose 7-phosphate, 3,4-dihydroxyphenyl-L-alanine, epinephrine, 5-aminovalericacid, L-malic acid, and adenosine diphosphate ribose.

**Table 8:** Metabolites and their corresponding metabolic pathways (YS-YB).

**Metabolite**	**Metabolic pathways**	**Fold-change**	***p***	**VIP**
N6,N6,N6-Trimethyl-L-lysine	Lysine degradation (map00310)	0.373	0.001	1.817
Palmitoleic acid	Fatty acid biosynthesis (map00061)	1.550	0.004	1.228
Taxifolin	Flavonoid biosynthesis (map00941)	0.474	0.009	1.392
Adenosine 5'-monophosphate	Purine metabolism (map00230); cGMP-PKG signaling pathway (map04022); cAMP signaling pathway (map04024); mTOR signaling pathway (map04150); PI3K-Akt signaling pathway (map04151); AMPK signaling pathway (map04152); Regulation of lipolysis in adipocytes (map04923); Renin secretion (map04924)	0.315	0.009	1.853
Inosine 5'-Monophosphate	Purine metabolism (map00230)	0.228	0.016	2.239
Trigonelline	Nicotinate and nicotinamide metabolism (map00760)	0.502	0.018	1.591
N6-Acetyl-L-lysine	Lysine degradation (map00310)	0.642	0.018	1.935
Guanosine monophosphate	Purine metabolism (map00230); cGMP-PKG signaling pathway (map04022)	0.540	0.019	1.626
Cortisol	Steroid hormone biosynthesis (map00140); Cortisol synthesis and secretion (map04927); Bile secretion (map04976)	2.477	0.022	1.069
Spermidine	Arginine and proline metabolism (map00330); beta-Alanine metabolism (map00410); Glutathione metabolism (map00480); ABC transporters (map02010); Bile secretion (map04976)	0.366	0.022	1.880
Traumatic acid	alpha-Linolenic acid metabolism (map00592)	0.498	0.024	1.497
Xanthosine	Purine metabolism (map00230)	0.624	0.035	1.562
Spermine	Arginine and proline metabolism (map00330); beta-Alanine metabolism (map00410); Glutathione metabolism (map00480); Bile secretion (map04976)	0.173	0.043	1.771
7α-Hydroxytestosterone	Steroid hormone biosynthesis (map00140)	0.515	0.048	1.510
D-Ribulose 5-phosphate	Pentose phosphate pathway (map00030); Pentose and glucuronate interconversions (map00040); Riboflavin metabolism (map00740); Vitamin B6 metabolism (map00750); Biosynthesis of amino acids (map01230)	0.258	< 0.001	2.595
L-Histidine	Histidine metabolism (map00340); beta-Alanine metabolism (map00410); Aminoacyl-tRNA biosynthesis (map00970); Biosynthesis of amino acids (map01230); ABC transporters (map02010); Protein digestion and absorption (map04974)	0.413	0.005	1.413
D-Sedoheptulose 7-phosphate	Pentose phosphate pathway (map00030); Biosynthesis of amino acids (map01230)	0.287	0.006	2.502
Prostaglandin J2	Arachidonic acid metabolism (map00590)	3.220	0.011	1.725
(9Z,11E,15Z)-(13S)-Hydroxyoctadeca-9,11,15-trienoate	alpha-Linolenic acid metabolism (map00592)	1.906	0.018	1.246
3,4-Dihydroxy-L-phenylalanine	Tyrosine metabolism (map00350)	0.423	0.025	1.925
L-Adrenaline	Tyrosine metabolism (map00350); cAMP signaling pathway (map04024); Regulation of lipolysis in adipocytes (map04923); Renin secretion (map04924)	0.641	0.025	1.545
5-Aminovaleric acid	Lysine degradation (map00310); Arginine and proline metabolism (map00330)	0.480	0.033	1.831
L-Malate	Citrate cycle (TCA cycle) (map00020); Pyruvate metabolism (map00620); Glyoxylate and dicarboxylate metabolism (map00630)	0.598	0.0335	1.563
Ascorbic acid	Ascorbate and aldarate metabolism (map00053); HIF-1 signaling pathway (map04066); Vitamin digestion and absorption (map04977)	2.097	0.0456	1.5296
Adenosine diphosphate ribose	Purine metabolism (map00230)	0.215	0.046	2.226

Map is the number of reference path diagrams in KGGE.

#### 3.5.3. Annotation of differential metabolic pathways in ruminant fluids

**Figure 5:**
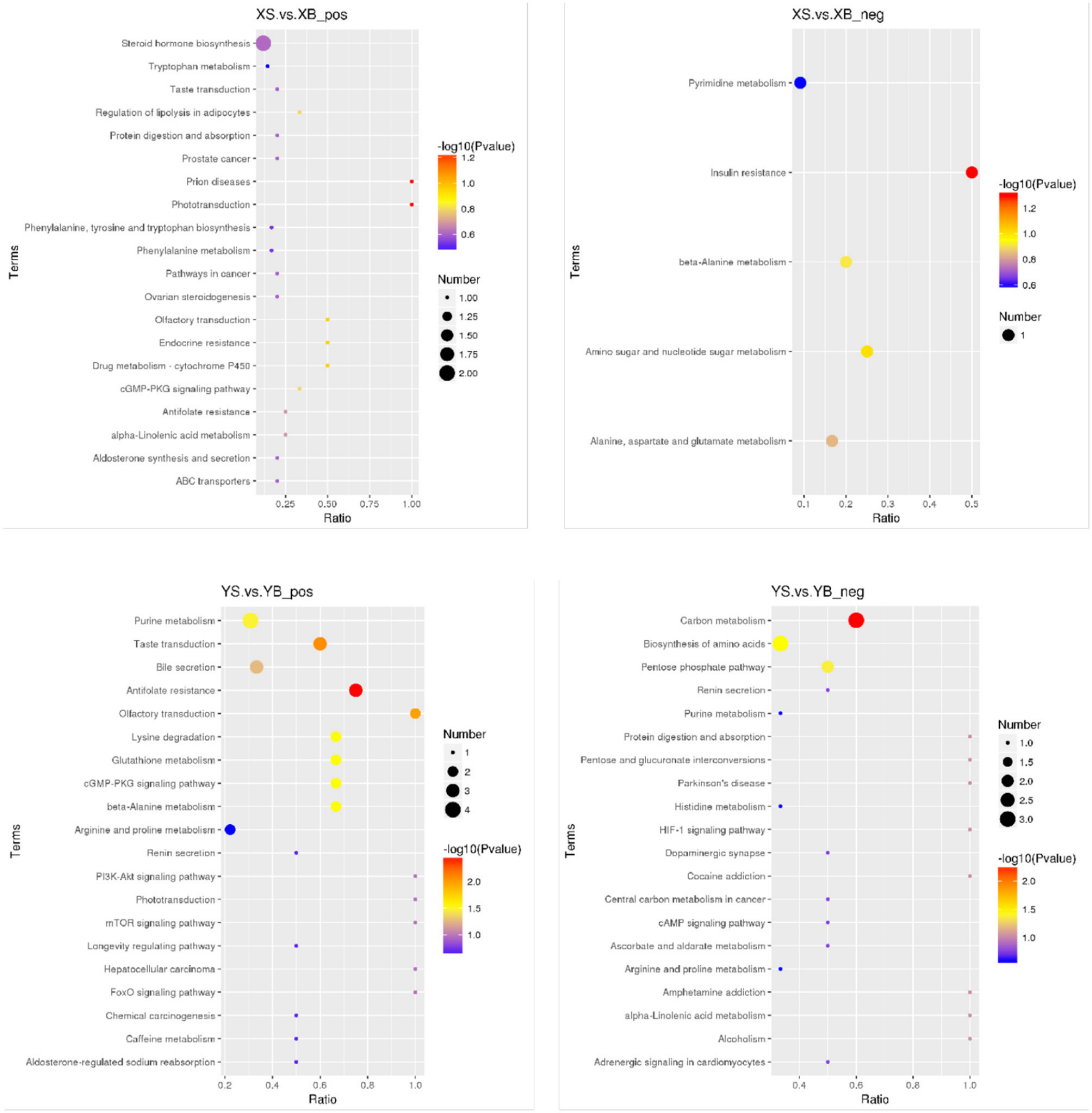
Bubble plot of KEGG enrichment in rumen fluid (positive and negative ion mode). The spot color represents the *p*-value in hypergeometric test, the size of the spot correlates with the number of the different metabolites in the pathway.

As shown in Figure 5, the results from the KEGG pathway enrichment for biological functions of differential metabolites are shown in the bubble chart. Compared to the XS group, the XB group has a higher enrichment in the insulin resistance pathways. Compared to the YS group, the YB group contains higher enriched pathways including lysine degradation, β-alanine metabolism, glutathione metabolism, the cyclic guanosine monophosphate-protein kinase G (cGMP-PKG) signaling pathway, purine metabolism, carbon metabolism, biosynthesis of amino acids, and the pentose phosphate pathway.

## 4. Discussion

In this study, phylogenetic analysis was conducted to determine the relationship between rumen microbial communities and the host animals. The results showed that, under the same growth environment and the same diet, different microbial structures were found in sheep and goats which belong to two different genera but under the same family. Previous studies on the interaction between host and microbial communities indicated that stomach and intestine microbiota are composed of many species and the community structure is influenced by the genetic background of the polygenic hosts. Different strains of the same species differed significantly in their environmental adaptations and digestive tract microbial composition. There is a clear genetic mixture between Wenchang and Linden chickens, which reveals the difference in adaptability between the two strains in tropical and cold environments from a genomic perspective (12). One of our previous study and other similar studies conclude that microbes residing in the animal digestion system differ among individual animals belonging to the same varieties or from separate varieties or species. An increasing number of studies have pointed out that the rumen microbial population is affected directly or indirectly by the genetic background of the ruminant hosts.

A whole-genome study on cows (*n* = 586) identified 61,974 single nucleotide polymorphisms (SNPs), together with the corresponding rumen bacterial communities, especially those with stronger heritability. It was found that the association between bacterial communities and host SNPs affected the selective uptake of rumen volatile lipid acids; the bacteria increased the energy utilization efficiency of the host. This study confirmed that the genetics of the host affect the rumen microbial communities, which in turn affect the phenotype of the host (13).

A recent study shows that the paternal species affected the type of microbiota which in turn affected the host metabolic activities (14). Studies on rumen fluid from wild yak and domesticated cows revealed different microbial communities in the rumen of the two animals. In the wild yak, a large number of unknown bacteria were identified (15). Under the same condition, studies on rumen microbial of two cow varieties also showed that the abundance and diversity of bacteria differed between the two cow varieties. All these results indicate that the host genetic background has a significant influence on the abundance of rumen bacteria (16). More studies indicate that, depending on the genetic background of host animals, they can have different levels of influence on the abundance of archaea or even the expression of microbial genes (17). However, it is not clear how the genotypes of the host animals affect the microbiota in the rumen.

In this study, rumen fluid samples were collected from two species of animals. Analysis of rumen microbiota revealed that different hosts contained largely similar compositions of bacterial communities. In the rumen microbiota, bacteria are mainly composed of Bacteroidetes, Firmicutes, and Proteobacteria. The Archaea is mainly Euryarchaeota from the genus of *Prevotella, Bacteroides*, and *Selenomona*. Our results are consistent with previous studies on the predominant microflora (18, 19). In domesticated and wild North American ruminant animals, the rumen-predominant microbial communities include Bacteroidete and Firmicutes (20). In the grazing Nellore cow, rumen, Firmicutes, Bacteroidetes, Proteobacteria, and Fibrobacteres are the predominant species, which account for 50% of total rumen bacterial phyla (21).

The Gram-positive Firmicutes bacteria are composed of species that can consume fermentable carbohydrates to obtain energy; its higher relative abundance may be related to the high starch content in the animal feed containing a high ratio of cereal. In the rumen, the methane-producing bacteria belong to Methanobrevibacter (22). The rumen microbial communities also have a complex and synergistic and antagonistic relationship with the host. As shown in the phylogenetic tree, during the establishment period, some very closely related bacterial strains can colonize ahead of the rest of the microbial species (23). The rumen core microbiota truly exists, but the relative abundance levels of the composite communities vary significantly among different species of host animals (24).

During the X and Y phases, Small-tail sheep and Boer goats were fed the same diet, but the microbiota differed between the two species; especially, there are significant differences in the abundance level of Proteobacteria, γ-proteobacteria, Aeromonadales, and Succinivibrionaceae. These results indicate that rumen microbiota is not completely affected by the diet, the growth of some microbial species is specific for the host species. Some members in Succinivibrionaceae utilize H2 to produce Succinate, which is then converted to propanoic acid. Methanogens also use H3 to produce methane at the expense of energy. The metabolism of propionate in the rumen will not produce methane. Succinivibrionaceae is known to compete for H with methane-producing bacteria to reduce the production of methane, so the host will have a better energy use efficiency (25). Pope et al. reported that, in *Macropus eugenii*, the methane production per unit of digestible energy is one-fifth of the ruminant animals, and its rumen is enriched with Succinivibrionaceae, possibly due to low methane production (26).

The microbiota of ruminant animals can be influenced by the genes of the host. In time, the inter-specific differences may widen even under the same feeding conditions, and aging is another factor affecting ruminant microbiomes. Continuous tracking studies on rumen microbial communities of 1-day to 2-year-old dairy cows found that, as cows grew up, the rumen microbial diversity and the similarity within each population increased while the bacterial communities gradually became stable (27).

These studies indicate that, in the digestive system of mammalian animals, it is possible that some factors may drive the establishment of stomach and intestine microbial communities. There are very few inter-specific differences in the index of rumen fermentation and the production of Propylene. There is no clear difference in the type of rumen fermentation among the different treatments conducted in this study. In the YB group, the rumen fluid microbial sequences were enriched into pathways including lysine degradation, metabolism of β-alanine and glutathione, the cGMP-PKG signal pathway, purine metabolism, amino acid biosynthesis, and the pentose phosphate pathway. Differential accumulated metabolites are trimethyl lysine, adenylic acid, 5'-inosinic acid, N6-acetyl-L-lysine, guanine, spermidine, xanthosine, spermine, D-ribofuranose, L-histidine, and D-sedoheptulose 7-phosphate.

## 5. Conclusion

Rumen microbial community is affected by the host. Different microbial structures were identified in sheep and goats. The Small-tail Han sheep had a higher feed utilization efficiency than the Boer goats. Bacteria in the Succinivibrionaceae family could be the microbial community that is responsible for this phenomenon. Additionally, rumen fluid metabolites are also affected by the host species. During the concentrate feed/crude food 5:5 diet, it was found that Boer goat rumen fluid contained the highest amino acid and nucleotide metabolic activities.

## Data availability statement

The datasets presented in this study can be found in online repositories. The name of the repository and accession number can be found below: NCBI; PRJNA881241.

## Ethics statement

The animal study was reviewed and approved by SDAUA-2022-67.

## Author contributions

XL, LJ, QC, and YJ: collection and assembly of data, data analysis and interpretation, and writing the article. QH, YW, and ZH: data analysis and interpretation. ZW: research concept and design. All authors contributed to the article and approved the submitted version.
